# Multi-level spatio-relational segformer (MLSRS-SegFormer): A novel vision transformer with adaptive spatial induction and dynamic positional encoding

**DOI:** 10.1016/j.mex.2025.103693

**Published:** 2025-10-29

**Authors:** Inda Rusdia Sofiani, Hadi Suyono, Erni Yudaningtyas, Fitri Utaminingrum

**Affiliations:** a Student of Doctoral Degree, Department of Electrical Engineering, Brawijaya University, Malang 65145, Indonesia; b Department of Vocational Education- Electronic Engineering Technology, Muhammadiyah University Malang, 65144, Indonesia; c Department of Electrical Engineering, Faculty of Engineering, Universitas Brawijaya, Malang 65145, Indonesia; d Department of Computer Science, Faculty of Computer Science, Brawijaya University, Malang 65145, Indonesia

**Keywords:** Medical Image Segmentation, Vision Transformer, Deep Learning, Hausdorff Bias, Relative Positional Encoding, Semantic Segmentation, Cervicography

## Abstract

Medical image segmentation is foundational to precision medicine. However, state-of-the-art Vision Transformers (ViTs) inherently suffer from a critical trade-off between comprehensive global contextualization and robust local boundary discrimination, especially in high-variance clinical data. This deficit necessitates a novel architecture. This study introduces the Multi-Level Spatio-Relational SegFormer (MLSRS-SegFormer), a novel vision transformer architecture designed to significantly enhance semantic segmentation through adaptive spatial induction strategies, dynamic positional encoding, and refined local context learning.

Our proposed Multi-Level Spatio-Relational SegFormer (MLSRS-SegFormer) model demonstrates significant architectural innovation, superior performance in comparative experiments, and robust validation for clinical applications, as summarized in the following key points:

• MLSRS-SegFormer integrates three clear and novel contributions beyond standard SegFormer: (1) **Adaptive Patch Weighting** in PatchEmbedding for dynamic feature induction, (2) **Hausdorff-bias Attention** for explicit spatial prioritization, and (3) **Relative Positional Encoding (RPE)** for nuanced and adaptive spatial relationship understanding.

• Comparative experiments reveal MLSRS-SegFormer's superior performance with consistent gains in segmentation accuracy, achieving the highest mIoU (0.968) and mDSC (0.980). Crucially for clinical applications, the model also demonstrates the lowest **HD95 (1.1668)**, which validates its **exceptional boundary precision**.

• Bland-Altman analyses further confirm its near-zero systematic bias and remarkable consistency in area and boundary delineation, providing robust and highly accurate segmentation vital for clinical applications despite a longer inference time.

## Graphical abstract

**Figure:**
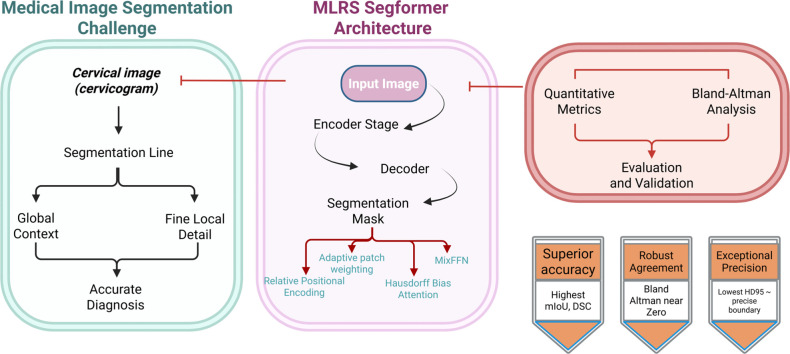

## Specifications table

**Table:** 

Subject area	Engineering
**More specific subject area**	*specifically, the subject is medical image semantic segmentation, with a focus on developing an innovative hybrid Vision Transformer architecture for accurate segmentation, particularly for cervical images in VIA screening procedures*
**Name of your method**	*Multi-Level Spatio-Relational SegFormer (MLSRS-SegFormer)*
**Name and reference of original method**	*S. Nurmaini et al., “Robust assessment of cervical precancerous lesions from pre- and post-acetic acid cervicography by combining deep learning and medical guidelines,” Informatics Med. Unlocked, vol. 52, no. June 2024, p. 101609, 2025, doi: 10.1016/j.imu.2024.101609.* *S. Xiang, “Colonic Polyp Segmentation Algorithm Based on Improved SegFormer Network,” 2023 3rd Int. Conf. Comput. Control Robot., pp. 101–105, 2023, doi: 10.1109/ICCCR56747.2023.10194105* *J. Li, Q. Xu, and X. He, “CFFormer : Cross CNN-Transformer channel attention and spatial feature fusion for improved segmentation of heterogeneous medical images,” Expert Syst. Appl., vol. 295, no. June 2025, p. 128835, 2026, doi: 10.1016/j.eswa.2025.128835.* *D. Dhinakaran, L. Srinivasan, S. E. Raja, K. Valarmathi, and M. Gomathy, “MethodsX Synergistic feature selection and distributed classification framework for high-dimensional medical data analysis,” MethodsX, vol. 14, no. December 2024, p. 103219, 2025, doi: 10.1016/j.mex.2025.103219.*
**Resource availability**	*Dataset: The primary dataset utilized in this study was meticulously collected from Dr. Mohammad Hoesin General Hospital in Palembang, Indonesia, during Visual Inspection with Acetic Acid (VIA) screening procedures in 2023 (Nurmaini et al., 2025). This comprehensive dataset encompasses approximately 3000 subjects with cervical images. Validation was also performed against an external public dataset from the International Agency for Research on Cancer (IARC).* *Hardware and Software: All experiments were conducted on a computational system featuring an Intel(R) Core(TM) i5-14400F processor, an NVIDIA GeForce RTX 4090 GPU with 24 GB of VRAM, a B760M K V2 DDR4 motherboard, and running on Windows 11. The development and execution environment was Spyder via Anaconda Integrated Development Environment (IDE), with models implemented and trained using the TensorFlow 3.9 programming framework.*

## Background

Medical image segmentation is fundamental for accurate disease diagnosis, pre-surgical planning, and post-interventional assessment. However, it faces complex challenges: anatomical variability, ambiguous boundaries, image noise, and diverse modalities. Addressing these is paramount for clinical practice and research.

While traditional methods (e.g., thresholding, region growing) were often insufficient, Convolutional Neural Networks (CNNs) like U-Net revolutionized medical image segmentation through powerful local feature extraction. However, their restricted receptive fields limit capturing long-range dependencies and global context, leading to suboptimal performance for large or dispersed objects. Vision Transformers (ViTs), adapted from Natural Language Processing, then excelled in global context and long-range dependency modeling. Yet, pure ViT deployment in medical imaging faces challenges: extensive dataset reliance, high computational costs, and difficulty preserving fine local details crucial for precise segmentation.

Consequently, hybrid CNN-Transformer architectures combine CNNs local feature extraction with Transformers global context and long-range dependency modeling. While promising in medical image segmentation [1], optimizing the balance of local and global features and adaptively capturing complex spatial relationships and relative positional information remains challenging, particularly for precise boundary delineation, minute lesions, or extreme object variations.

While state-of-the-art architectures like SegFormer excel in general semantic segmentation, their direct medical imaging application is constrained by limitations in capturing fine spatial details and nuanced relative patch positions. Standard SegFormer may not fully optimize long-range dependency modeling with crucial local spatial information, especially for ambiguous boundaries or small objects. This identifies a research gap: a Vision Transformer explicitly integrating multi-level spatial induction and adaptive relative positional understanding for enhanced medical image segmentation accuracy and efficiency.

To decisively address this critical deficit where existing Vision Transformers struggle to balance comprehensive global context and fine-grained local boundary accuracy, we propose the Multi-Level Spatio-Relational SegFormer (MLSRS-SegFormer), an innovative architecture engineered for high-precision medical image segmentation. The MLSRS-SegFormer fundamentally integrates dynamic induction and multi-level spatial weighting strategies to bolster both positional and spatial understanding. This is achieved through three core innovations: first, the introduction of an **Adaptive Patch Weighting (APW)** module during PatchEmbedding, which dynamically prioritizes feature importance; second, the development of a **Hausdorff-bias Attention (HBA)** layer that enforces explicit spatial bias via an auxiliary Hausdorff distance loss, enhancing boundary sensitivity; and third, the implementation of **Dynamic Spatio-Relational Encoding** utilizing Relative Positional Encoding (RPE) coupled with local feature reinforcement (via DepthwiseConv in MixFFN) to mitigate positional information loss across multi-scale feature maps, leading to demonstrably superior performance in challenging segmentation tasks.

### Method details

This section provides a comprehensive overview of the experimental design, beginning with the architecture of our proposed Multi-Level Spatio-Relational SegFormer (MLSRS-SegFormer). It then details the datasets, training configurations, and evaluation metrics used to validate its performance against other state-of-the-art models. The central aim of this study was to develop and assess an advanced vision transformer tailored for semantic segmentation, with a specific focus on enhancing its capacity to capture intricate spatial details and contextual relationships through the integration of multi-level spatial induction strategies and adaptive positional understanding.

The recognition of the complementary strengths of CNNs and ViTs has spurred the development of hybrid architectures, aiming to mitigate their individual weaknesses by leveraging their combined capabilities [1]. These hybrid models broadly fall into several categories: many integrate Transformers into encoder-decoder frameworks, such as MaxViT-UNet, which employs MaxViT blocks and multi-axis self-attention to boost accuracy and generalization [2], UNestFormer, utilizing nested Transformers for enhanced decoders and skip connections [3], Enhanced-TransUNet, which improves U-Net with Transformer and refined skip features [4], ETUNet for efficient 3D brain tumor segmentation [5], STCNet for COVID-19 lesion segmentation [6], and TransUNet+, redesigning skip connections for feature enhancement [7]. Other hybrid approaches emphasize multi-scale feature fusion, including MixFormers CNN–Transformer hybrid encoder with spatial-aware multi-scale fusion [8], HTC-Nets balance of local and global information using Trident Multi-layer Fusion and United Attention [9], MFMSNets integration of multi-frequency analysis and multi-scale fusion [10], BiFTransNets unified CNN-Transformer with BiFusion module for GI segmentation [11], DBFFTs fusion of spatial and frequency domain features [12], and TSCA-Nets effective extraction and fusion of multi-scale global features [13]. Additionally, attention-enhanced hybrids, such as HAU-Net with its L-G transformer blocks and CAB for multi-scale dependencies [14], HMDAs Multi-scale Deformable Attention [15], TransUMobileNets Multi-Channel Attention Fusion [16], DMSA-UNets efficient Dual Multi-Scale Attention [17], PFormers innovative P-attention for 3D segmentation [18], and ADRNets Mixed Attention Transformer for brain MRI registration [19], all signify concerted efforts to refine feature integration and attention mechanisms.

Despite significant advancements, current hybrid architectures continue to face challenges in optimizing feature integration, preserving ultra-fine boundary details, and adaptively capturing complex spatial relationships across various scales and positions. Some models, for instance, still contend with the loss of global information between modules [20], while others struggle to effectively capture long-range dependencies for complex tumor shapes [10]. Consequently, the importance of precise spatial context and robust positional understanding has come to the forefront. Furthermore, the role of positional encodings in Transformers, evolving from absolute to more adaptive relative positional encoding (RPE), is critical. While absolute positional encodings might limit generalization, RPE allows models to adaptively learn and weight relative spatial relationships, offering a more dynamic and flexible contextual understanding, a concept also seen in dual position encoding methods [21].

Building upon this extensive body of work, a critical research gap persists where existing state-of-the-art models, including the standard SegFormer architecture, still grapple with achieving optimal integration of multi-level spatial understanding and adaptive relative positional relationships, particularly crucial for the nuanced demands of medical image segmentation. To precisely address this gap, this study introduces the Multi-Level Spatio-Relational SegFormer (MLSRS-SegFormer), which uniquely fills this void through a comprehensive integration of innovative strategies. This includes adaptive patch weighting within the PatchEmbedding layer to establish an enhanced foundational spatial understanding, the pioneering application of a Hausdorff-bias-based Attention mechanism that provides an explicit and measurable spatial induction, the reinforcement of local spatial context learning through DepthwiseConv2D within the MixFFN layer, and the crucial integration of Relative Positional Encoding (RPE) to foster a more dynamic and adaptive comprehension of relative spatial relationships among tokens. The combination of these multi-faceted strategies, not previously explored collectively in the context of Vision Transformers for medical image segmentation, constitutes the core novelty and primary contribution of our research.

#### Overview methodology

To facilitate comprehencive understanding of this research, this section provides an overview of the experimental design. It introduces the proposed Multi-Level Spatio-Relational SegFormer (MLSRS-SegFormer) architecture, and also delves into the datasets used, training configurations, and chosen evaluation metrics. At its heart, this study aimed to develop and assess an advanced vision transformer specifically for semantic segmentation. The particular focus was on enhancing its ability to capture intricate spatial details and truly understand contextual relationships. This was achieved by thoughtfully integrating multi-level spatial induction strategies and a deeper grasp of relative positions. It is expected that the MLSRS-SegFormer will demonstrate more accurate and efficient segmentation performance when compared to existing state-of-the-art models.

The experimental design is comparative, rigorously evaluating MLSRS-SegFormer against 7 other semantic segmentation model architectures. All experiments were conducted on a computer featuring an Intel(R) Core(TM) i5-14400F processor (2.5 GHz, 10 cores, 16 logical processors) and a B760M K V2 DDR4 motherboard. The core of this methodology lies in MLSRS-SegFormer, an evolution of the SegFormer architecture engineered for heightened spatial awareness via multi-level spatial induction. It enhances PatchEmbedding by initially weighting input patches through Conv2D and LayerNormalization to form embed_dim vector tokens. Within each TransformerEncoderLayer, HausdorffMultiHeadAttention explicitly integrates a hausdorff_bias computed from Euclidean distances, prioritizing nearby spatial relationships for precise boundary delineation. Local spatial context is bolstered by MixFFNs use of DepthwiseConv2D within TransformerEncoderLayer, allowing independent spatial processing per feature channel, adding a learnable induction layer. Crucially, Relative Positional Encoding (RPE) is integrated into the self-attention, enabling adaptive learning and weighting of relative positional relationships between tokens, complementing the static Hausdorff bias with dynamic, flexible contextual spatial understanding.

The dataset for this research was sourced from the appended journal [22]. Training involved 600 epochs, with 1000 steps_per_epoch and 200 validation_steps. Callback mechanisms included ReduceLROnPlateau, which decreased the learning rate by a factor of 0.5 if val_loss did not improve for 15 epochs (patience=15) down to a minimum of 1e-15, and EarlyStopping, which halted training after 35 epochs of no val_loss improvement (patience=35) while restoring the best model weights. While specific optimizer and initial learning rate were not explicitly stated, common choices for deep learning, such as Adam or AdamW with initial rates like 1e-3 or 1e-4, are typically employed. Model performance was quantitatively assessed using mean Jaccard (mIoU), mean Dice Similarity Coefficient (mDSC), mean F1-score, mean HD95, Total Parameters, Average Prediction Time per Image (s), and Validation Loss (val_loss). Four types of Bland Altman plots were generated for Area, HD95, IoU, DSC, and F1 metrics.

#### Proposed MLSRS-SegFormer architecture

**Fig. 1:**
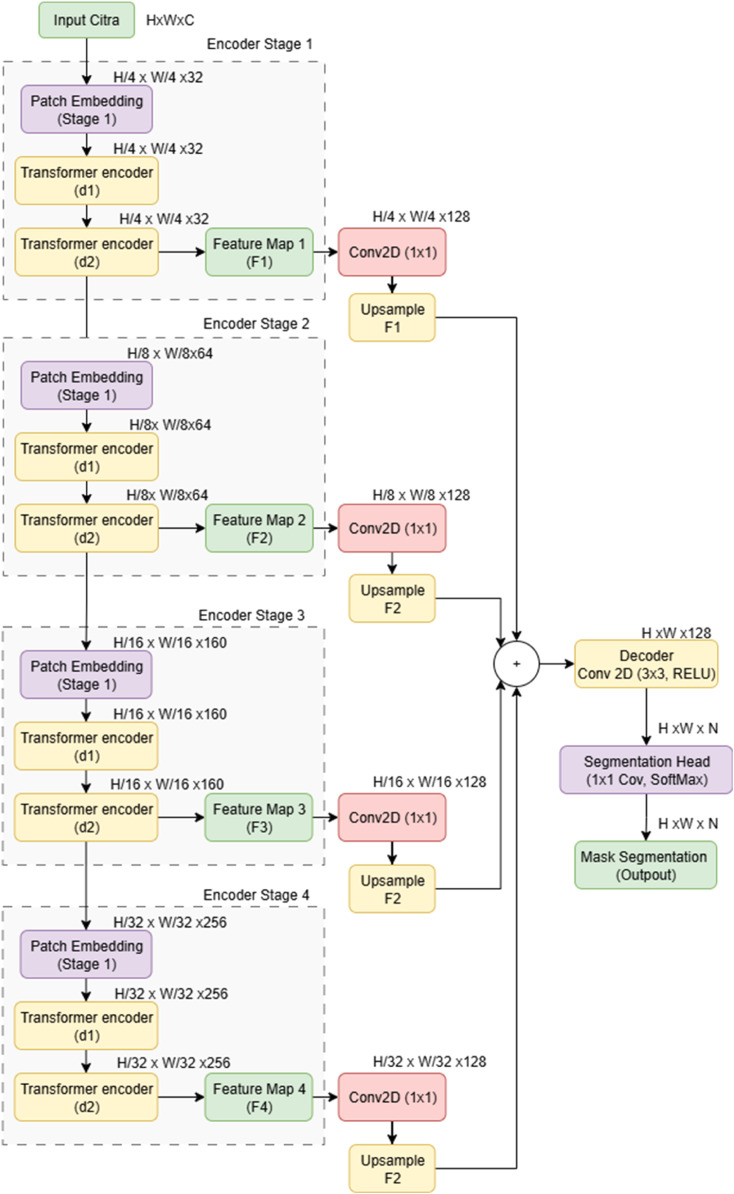
Overall architecture of the proposed MLSRS-SegFormer.

The proposed Multi-Level Spatio-Relational SegFormer (MLSRS-SegFormer) is meticulously designed as an advanced encoder-decoder architecture, specifically tailored for robust semantic segmentation in complex scenarios such as medical imaging. As depicted in Fig. 1, the architecture extends the foundational principles of SegFormer by leveraging a hierarchical Transformer-based encoder for efficient multi-scale feature extraction, complemented by a lightweight, yet highly effective, all-MLP decoder for generating precise segmentation masks. This integrated design aims to achieve an optimal balance between capturing extensive global contextual information and preserving intricate local spatial details, which is paramount for high-fidelity segmentation.

The architecture begins by taking in an input image, which then moves through several layered encoder stages. Inside each of these encoder stages, a PatchEmbedding layer is the first step, turning either the input image or the feature map from the step before into a stream of tokens. This transformation happens using a Conv2D operation that pushes these patches into a richer, more complex space, and then a LayerNormalization step helps stabilize things. Whats clever here is that this PatchEmbedding also includes a learnable patch_weight that can actually learn to adjust how important each patch is, getting them ready as vectorized tokens for whatever comes next.

After the PatchEmbedding, each encoder stage has several TransformerEncoderLayer modules. These layers are really the brains of the encoder, built carefully to Fig. out complex connections between features. They come with some neat improvements, like a HausdorffMultiHeadAttention mechanism. This one specifically adds a bias based on how far things are from each other (Euclidean distance), helping it focus on close-by spatial relationships. Plus, theres RelativePositionBias2D, which lets the model dynamically learn about relative positions. On top of that, the MixFFN within these layers uses DepthwiseConv2D to make sure it really understands local spatial details. Finally, the output features from each encoder stage get turned into multi-scale feature maps, and then gradually sent over to the decoder.

#### Adaptive patch weighting in PatchEmbedding

Input images first enter the Transformer encoder through the PatchEmbedding layer. This is where raw image patches are converted into sequences of learnable tokens. This initial step is vital because it establishes the basic spatial understanding that will be used across the entire Multi-Level Spatio-Relational SegFormer (MLSRS-SegFormer) architecture. When an image is received, a Conv2D layer (labeled as self.proj) is applied. Its kernel size and stride are set according to how the patches are conFig.d, essentially projecting the image patches into a richer, higher-dimensional embedding space. This convolutional process naturally assigns initial weights based on local patterns, effectively transforming the patches into feature maps from which the token vectors are eventually derived.

Once the convolutional projection is done, the resulting feature map is flattened and turned into a sequence of tokens. Now, a really neat part about this PatchEmbedding layer is how it includes an adaptive patch_weight mechanism. This self.patch_weight is a parameter that the model can learn from scratch; it begins as ones and matches the number of patches. This clever setup lets the model dynamically decide which patches are more or less important.

This adaptive weighting then gets applied to the flattened patch embeddings, working on each element individually as illustrated in Equation (1).

(1) $$X_{\text{flat}\_\text{weighted}\;}=X_{\text{flat}\;}⊙W_{\text{patch}\;}$$

Where $X_{\text{flat}\;}$represents the flattened patch embeddings after convolutional projection, and $W_{\text{patch}\;}$ denotes the learnable adaptive patch weights. The symbol ⊙indicates element-wise multiplication. This weighting mechanism provides a flexible way to enhance or suppress information from different spatial locations at the very first stage of tokenization, improving the models sensitivity to important regions.

Finally, the weighted patch embeddings are subjected to LayerNormalization (self.norm) to stabilize the training process and improve model performance. Layer Normalization operates independently on each sample across its feature dimensions, standardizing the activations. The output of this normalization step, $X_{\text{norm}\;}$, is computed as per Equation (2):

(2) $$X_{\text{norm}\;}=\gamma ⊙\frac{X_{\text{natu}-\text{weighted}\;}-\mu }{\sqrt{\sigma^{2}+\epsilon }}+\beta \;\;$$

Here, μ and $\sigma^{2}$ are the mean and variance of $X_{\text{flat}\_\text{weighted}\;}$ across the feature dimension for each sample, respectively. γ and β are learnable scaling and shifting parameters, and ϵ is a small constant to prevent division by zero. The resulting $X_{\text{norm}\;}$ tokens, along with their spatial dimensions (H and W), are then passed to the subsequent Transformer encoder layers. This adaptive patch weighting, coupled with robust normalization, ensures that the initial token representations are finely tuned for spatial awareness and prepared for deeper contextual processing within the MLSRS-SegFormer.

#### Hausdorff-bias-based multi-head attention

**Fig. 2:**
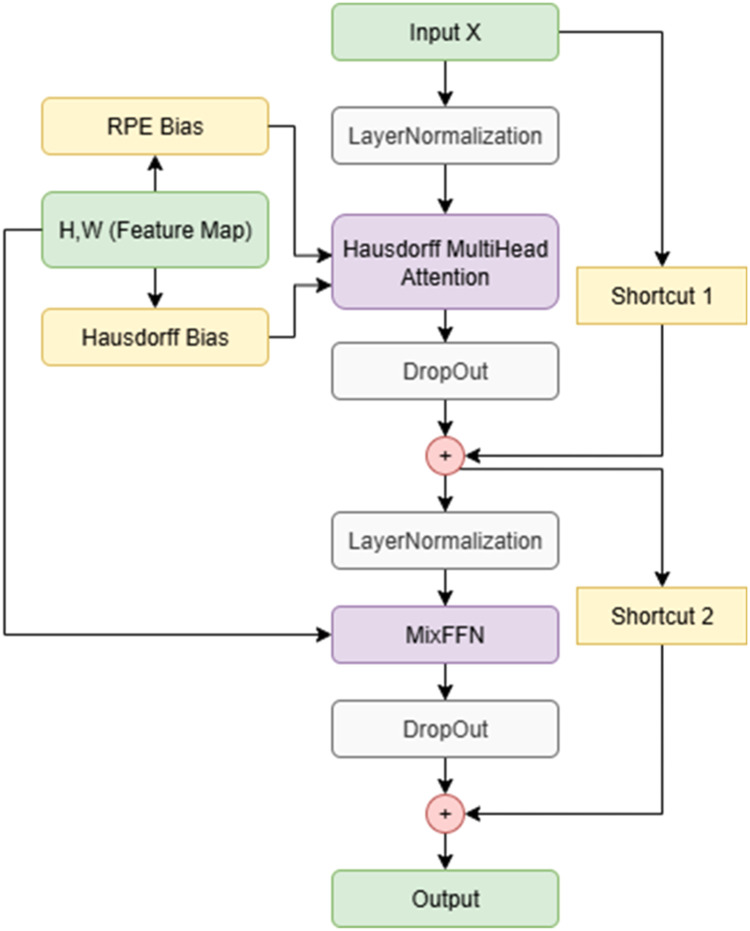
Architecture of the relative positioning encoding of MLSRS-SegFormer.

The HausdorffMultiHeadAttention layer is a critical enhancement within the TransformerEncoderLayer of MLSRS-SegFormer, specifically designed to inject an explicit spatial prior into the self-attention mechanism as shown in Fig. 2. While standard Multi-Head Attention effectively captures global dependencies by computing attention scores between all tokens, it lacks an inherent understanding of their spatial proximity, which is vital for fine-grained segmentation tasks. To address this, our modified attention mechanism augments the traditional attention scores with a pre-computed Hausdorff bias.

This hausdorff_bias is derived from the Euclidean distances between all possible pairs of patch centroids within the feature map grid. For a feature map of dimensions H×W, resulting in N=H×W patches, the Euclidean distance $d(p_{i},p_{j})\;$between any two patches $p_{i}$and $p_{j}$ is calculated. This distance is then normalized and negated to form the bias matrix $B^{\text{Hausdorff}\;},\;$as described in Equation (3):

(3) $$B_{ij}^{\text{Hausdorff}\;}=-\frac{d\left(p_{i},p_{j}\right)}{\text{max}\left(D\right)+\epsilon }$$

Here, D represents the matrix of all pairwise Euclidean distances between patch centroids, max(D) is the maximum distance within this matrix, and ϵ is a small constant to ensure numerical stability. The negative sign ensures that smaller spatial distances (i.e., closer patches) result in larger positive biases, thereby implicitly increasing their attention weight.

During the forward pass of HausdorffMultiHeadAttention, the initial attention scores (S) are computed by the standard Multi-Head Attention module. Subsequently, if the hausdorff_bias is provided, it is added directly to these raw attention scores before the softmax operation, as formulated in Equation (4):

(4) $$S_{b,h,i,j}^{\prime }=S_{b,h,i,j}+B_{i,j}^{\text{Hausdorff}\;}$$

Where $S_{b,h,i,j}^{\prime }$ are the modified attention scores for batch b, head b, query patch i, and key patch i. This explicit addition of $B^{\text{Hausdorff}\;}$ (broadcast across batch and head dimensions) to the attention scores biases the model to prioritize relationships between spatially closer patches. This mechanism significantly enhances the models ability to maintain continuity and delineate boundaries more accurately, as it encourages attention to relevant neighboring regions, a crucial aspect often overlooked by purely global attention mechanisms. Following this, the attn_probs are calculated using a softmax function over $S^{\prime }$, effectively converting the scores into attention probabilities.

#### Local spatial context learning in MixFFN

Beyond the global contextual understanding facilitated by attention mechanisms, maintaining and enhancing local spatial context is crucial for high-fidelity segmentation, especially for fine details and boundaries in medical images. The MixFFN (Mixed Feed-Forward Network) layer within each TransformerEncoderLayer in MLSRS-SegFormer is specifically designed to address this by integrating a local spatial processing component.

The MixFFN first processes the input tokens x (which are typically normalized output from the attention sub-layer) through a fully connected layer (self.fc1) that expands the feature dimensionality, followed by a GELU activation function (self.act). This output u is then reshaped from a flattened sequence of tokens (Batch, HW, Expanded_Channels) back into a spatial format (Batch, H, W, Expanded_Channels), denoted as $U_{\text{spatial}\;}$. This reshaping allows for the application of convolutional operations.

The core of local spatial context learning in MixFFN is achieved through the application of a DepthwiseConv2D layer (self.dwconv). Unlike standard convolutions that operate across all input channels simultaneously, a depthwise convolution applies a single convolutional filter to each input channel independently. For an input feature map $U_{\text{spatial}\;}\;$with $C_{\text{exp}}\;$channels, the output $U_{\text{dw}}$ from the depthwise convolution can be represented for each channel c by Equation (5).

(5) $$U_{\mathrm{dw}}\left[b,i,j,c\right]=\sum k,lU_{\mathrm{spatial}}\left[b,i+k,j+l,c\right]\cdot K\left[k,l,c\right]$$

Where b is the batch index, i,j are spatial coordinates, c is the channel index, and K is the kernel of the DepthwiseConv2D layer. This operation, performed with a kernel_size=3 and padding=same, allows each channel to learn local spatial patterns without mixing information between different channels at this stage, thus enriching the token representation with localized contextual cues.

Following the depthwise convolution, the spatially processed feature map $U_{\text{dw}}$ is again flattened back into a sequence of tokens ($U_{\text{flat}\;}$) and then normalized using LayerNormalization (self.norm) to stabilize the feature distribution. Finally, a second fully connected layer (self.fc2) projects these normalized features back to the original embedding dimension (embed_dim), producing the final output out of the MixFFN. This strategic integration of DepthwiseConv2D within the MixFFN allows MLSRS-SegFormer to effectively capture detailed local spatial context, complementing the global feature learning of the attention mechanism and contributing significantly to the models ability to perform accurate segmentation, particularly for intricate structures and fine boundaries.

#### Integration of relative positional encoding (RPE)

Relative Positional Encoding (RPE) really sets MLSRS-SegFormer apart. It helps the model dynamically pick up on and highlight how different tokens relate to each other in terms of their position. While absolute positional encodings give static location information, RPE takes a different approach. Instead, it focuses on the relative distance between any two elements. This gives it a much more flexible and broader understanding of spatial relationships, especially when paired with direct spatial cues, like the Hausdorff bias. This kind of adaptive learning is absolutely essential for dealing with the varied object scales and positions often found in complex medical images.

The RelativePositionBias2D layer, instantiated within each TransformerEncoderLayer, is responsible for generating these dynamic positional biases. It maintains two sets of trainable parameters: self.relative_height and self.relative_width. These parameters represent learnable biases for relative vertical and horizontal displacements between any pair of patches. During the forward pass, for a given feature map of height H and width W, the layer calculates all pairwise relative coordinates Δy,Δx for every possible pair of patches. These relative coordinates are then used to look up the corresponding biases from the learned height and width bias tables. The combined RPE bias for a pair of patches $\left(p_{i},p_{j}\right)$ and a specific attention head k is given by Equation (6):

(6) $$B_{ij,k}^{\text{RPE}}=B_{\text{height}\;}\left[y_{i}-y_{j}+S_{y},k\right]+B_{\text{width}\;}\left[x_{i}-x_{j}+S_{x},k\right]\;\;$$

Where $B_{\text{height}\;}\;$and $B_{\text{width}\;}\;$are the learned height and width bias tables, respectively. $\left(y_{i},x_{i}\right)$ and $\left(y_{j},x_{j}\right)$ are the 2D coordinates of patch i and patch $j.S_{y}\;$and $S_{x}$are shifting constants (equal to max_relative_position - 1) used to map the relative differences into positive indices for array lookup. This calculated $B^{\text{RPE}}\;$is a tensor of shape [num_heads, $H^{*}w,H^{*}w$], where $H^{*}\;W$ is the sequence length (number of patches).

The generated rpe_bias is then passed to the HausdorffMultiHeadAttention module. This is where the RPE bias interacts with the attention scores. After the initial attention scores (S) are computed and augmented with the Hausdorff bias $B^{\text{Hausdorff}\;}$ as shown in Equation (4) from the previous section, leading to $S^{\prime }$), the RPE bias is directly added to these scores before the softmax operation. This final modification of the attention scores $\left(S^{\prime \prime }\right)$ is formulated in Equation (7):

(7) $$S_{b,h,i,j}^{\prime \prime }=S_{b,h,i,j}^{\prime }+B_{h,i,j}^{\text{RPE}}$$

Here, $S_{b,h,i,j}^{\prime \prime }$ represents the fully biased attention score for batch b, head h query patch i, and key patch j. The addition of $B^{\text{RPE}}$ (broadcast across the batch dimension) allows the attention mechanism to dynamically consider the relative spatial positioning between tokens, providing a more nuanced and adaptive weighting to their interactions. This multi-layered biasing strategy, combining static Hausdorff bias with dynamic RPE, is central to MLSRS-SegFormers enhanced spatial reasoning, enabling a more precise and contextually aware segmentation performance.

#### Dataset, hardware and software

The primary dataset for this study, collected from Dr. Mohammad Hoesin General Hospital in Palembang, 2023, consists of approximately 3000 cervical images from Visual Inspection with Acetic Acid (VIA) screenings [22]. This collection includes images pre- and post-acetic acid application, manually annotated by two oncologists for cervical, columnar, and lesion areas. Initial data comprised roughly 2700 normal and 300 abnormal cervicograms, with strict quality inclusion criteria [22]. To address the scarcity of abnormal samples and ensure model robustness, these were augmented from 261 to 521. Model generalizability was additionally tested against an external public dataset from the International Agency for Research on Cancer (IARC) [22].

Data preprocessing involved pixel normalization and resizing all images to 256×256 pixels. The dataset was then split into 80% for training, 10% for validation, and 10% for testing. A multi-faceted augmentation strategy included an initial super-resolution step (ESRGAN, BSRGAN) applied to images. Subsequent augmentations, synchronized with image masks, included random noise, spatial cropping, diverse rotations, horizontal/vertical flipping, scaling, and brightness/contrast adjustments. This holistic approach significantly bolstered the models ability to generalize. All experiments were conducted on a high-performance workstation running Windows 11 and utilizing the Spyder IDE (via Anaconda) with the TensorFlow 3.9 framework. The system was equipped with an Intel Core i5-14400F processor and a high-end **NVIDIA GeForce RTX 4090 GPU with 24 GB of VRAM** for accelerated deep learning computations.

#### Training configuration

The training process for MLSRS-SegFormer and its comparative models was meticulously conFig.d to ensure optimal performance and convergence. The global policy for numerical precision was set to float32 using tf.keras.mixed_precision.set_global_policy (float32), ensuring full precision computations. The model was compiled with the Adam optimizer, initialized with an initial learning rate of 1e-4 (0.0001) and a clipnorm of 1.0, and wrapped within a LossScaleOptimizer for stable training.

Our approach involved a composite loss function, specifically combining Dice Loss with Categorical Cross-Entropy (CCE). This particular combination was chosen to effectively manage segmentation tasks, allowing us to account for both accurate pixel-wise classification (thanks to CCE) and good spatial overlap (via Dice Loss). For the training itself, we ran the model for 600 epochs, with each epoch encompassing 1000 steps for the training data and 200 steps for validation. For the training configuration, we ran the model for 600 epochs, with each epoch encompassing 1000 training steps and 200 validation steps. Crucially, to accommodate the high memory requirements inherent to the transformer architecture and ensure stable training, a **consistent batch size of 1** was used for both training and validation data streams across all experiments..

To help guide the training process and keep the model from overfitting, we set up a few important callbacks. One was ReduceLROnPlateau, which would dynamically cut the learning rate by half if the validation loss didnt show improvement for 15 consecutive epochs, ensuring it wouldnt drop below a minimum of 1e-15. Another crucial callback, EarlyStopping, was put in place to monitor the validation loss; if no improvement was seen for 35 epochs, training would halt, and the model would revert to the weights that yielded the best validation loss. Additionally, a ModelCheckpoint callback was included, primarily to save the best model weights based on the validation loss throughout the training, making sure we retained the most performant version.

#### Comparative models

To truly gauge how effective and advanced our proposed Multi-Level Spatio-Relational SegFormer (MLSRS-SegFormer) is, we carried out a comprehensive comparison. We put it head-to-head with several other leading semantic segmentation architectures. This benchmark included specific SegFormer variants such as Segformer_Loss, Segformer Attention, and the Segformer B0 baseline [23], along with BifTransNet [11], PVT [24], and nnFormer [25]. A crucial part of this process was making sure every single one of these models was tested under identical experimental conditions and processing pipelines. This meant we meticulously kept everything consistent—from the hardware and software used, to the data preprocessing, augmentation strategies, and even the training configurations. This rigorous, standardized approach was vital for a fair and unbiased assessment of their individual segmentation capabilities. Ultimately, this allowed us to confidently highlight the unique advancements that MLSRS-SegFormer offers.

#### Quantitative metrics

the performance of all models was quantitatively evaluated using a comprehensive set of standard metrics. segmentation accuracy and overlap were assessed using the mean intersection over union (miou), mean dice similarity coefficient (mdsc), and f1-score. to specifically evaluate the precision of boundary delineation, which is critical in clinical applications, we utilized the 95th percentile hausdorff distance (hd95). additionally, model complexity and computational efficiency were measured by the total number of trainable parameters and the average inference time per image, respectively. and of course, in clinical settings, this level of exactness is absolutely crucial, since getting precise morphological measurements is often a really big deal.

#### statistical analysis

Key components of a Bland-Altman plot include the mean difference, which indicates any systematic bias between the two methods, and the Limits of Agreement (LoA), typically calculated as the mean difference ± 1.96 times the standard deviation of the differences [26],[27]. These LoA define the range within which 95% of the differences between the two methods are expected to lie, allowing for an assessment of the clinical or practical acceptability of the agreement [26]. However, it is crucial to consider the underlying assumptions: the Bland-Altman method assumes that the methods have similar and constant precision, and that any bias is constant (differential bias only) [28]. If these assumptions are violated (e.g., due to proportional bias or unequal variances), the plot can be misleading, potentially necessitating advanced adaptations, such as regressing differences on means or the use of repeated measurements to discern individual precision and true biases [28],[27].

### Method validation

#### Quantitative performance analysis

The comparative analysis of various semantic segmentation architectures, as summarized in Table 1, provides a comprehensive overview of their performance across key metrics. Generally, the SegFormer-based models demonstrate a strong performance, particularly in terms of segmentation accuracy, when compared to general Vision Transformer models like PVT and nnFormer. Among all evaluated architectures, the proposed Multi-Level Spatio-Relational SegFormer (MLSRS-SegFormer) consistently emerges as the top performer in terms of segmentation accuracy and boundary precision, albeit with a trade-off in inference speed.

**Table 1:** Overall performance summary of semantic segmentation models.

Model Architecture	Average Jaccard (mIoU)	Average Predicted DSC	Average Predicted F1	Average Predicted HD95	Total Parameters	Average Prediction /Image (s)	Average val_loss
Segformer B0 [23]	0.951	0.9682	0.7086	1.6708	4855460	0.121	0.02
BifTransNet [11]	0.8328	0.8798	0.6420	11.0897	4893924	0.094	0.107
nnFormer [25]	0.841	0.8849	0.6538	11.1454	8502116	0.093	0.105
PVT [24]	0.903	0.9353	0.6809	4.5871	8028516	0.094	0.05
**Multi-Level Spatio-Relational SegFormer (MLSRS)-ours**	**0.968**	**0.98**	**0.7176**	**1.1668**	**4862916**	**0.298**	**0.014**

Table 1 presents a comparative performance analysis of various deep learning models for a segmentation task, highlighting key metrics such as segmentation accuracy, boundary precision, model complexity, and inference speed. The models under comparison include Segformer B0 [2], BifTransNet [17], nnFormer [28], PVT [27], and the proposed Multi-Level Spatio-Relational SegFormer (MLSRS)-ours. In terms of segmentation accuracy, the MLSRS-ours model demonstrates superior performance across critical metrics. It achieves the highest Average Jaccard (mIoU) score of 0.968, indicating excellent overlap between predicted and ground truth segmentations.

Similarly, its Average Predicted DSC (Dice Similarity Coefficient) of 0.98 and Average Predicted F1 score of 0.7176 are the highest among all compared models, reinforcing its strong capability in accurate segmentation and balanced precision-recall. For boundary delineation, MLSRS-ours also excels with the lowest Average Predicted HD95 (Hausdorff Distance at 95th percentile) of 1.1668, suggesting more accurate and smoother boundaries compared to alternatives like BifTransNet (11.0897) and nnFormer (11.1454). Even against Segformer B0 (1.6708) and PVT (4.5871), the MLSRS-ours model shows a significant improvement in boundary precision.

Regarding model complexity, MLSRS-ours has 4,862,916 total parameters, which is comparable to Segformer B0 (4,855,460) and BifTransNet (4,893,924), and notably more efficient than nnFormer (8,502,116) and PVT (8,028,516).

However, this high performance comes with a trade-off in inference speed, as MLSRS-ours exhibits the longest Average Prediction per Image time at 0.298 seconds. This is notably slower than other models like BifTransNet (0.094 s), nnFormer (0.093 s), and PVT (0.094 s). Lastly, the lowest Average val_loss of 0.014 for MLSRS-ours signifies its robust training and generalization capabilities, outperforming all other models.

#### Convergence, stability, and indication of overfitting/underfitting

**Fig. 3:**
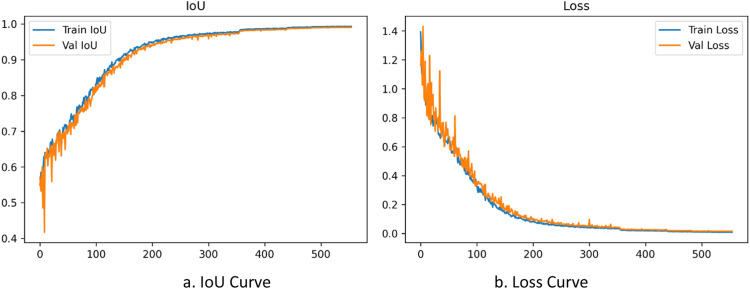
Curves of MSLRS model architecture.

The training dynamics of the MLSRS-SegFormer model, in terms of convergence, stability, and potential overfitting or underfitting, can be thoroughly analyzed by examining the Intersection over Union (IoU) and loss curves across both the training and validation datasets Fig. 3.

**E rror! Reference source not found.**a, displaying the IoU curves, illustrates a robust learning progression. Both the Train IoU and Val IoU metrics demonstrate a rapid ascent during the initial ∼150 epochs, indicating efficient early-stage learning. The rate of increase then becomes more gradual, though it continues to progress steadily. Both curves ultimately approach a near-perfect score of 1.0 by the 600th epoch. This parallel upward movement, where the Val IoU closely tracked Train IoU, is a strong sign of robust convergence and effective generalization. The narrow, consistent gap between the two curves throughout the training process indicates that the model learned efficiently from the training data and successfully generalized this understanding to unseen validation data, without clear signs of overfitting. Minor initial fluctuations in Val IoU during the very early stages are common and settled quickly as the models weights began to consolidate.

Observing the loss curves in **E rror! Reference source not found.**b, alongside the IoU trends, provides further confirmation of the models stable and effective learning. During the first 100-150 epochs, both the Train Loss and Val Loss dropped sharply, indicating the model quickly reduced errors and adjusted to the dataset. After this initial steep drop, loss values continued to fall, though more gradually, eventually nearing zero. Crucially, the Val Loss curve remained very close to the Train Loss curve throughout training, keeping a narrow and consistent gap. This close relationship between training and validation losses is a clear sign of the models high stability and its strong generalization ability. It strongly suggests there was no significant overfitting (where validation loss would rise) or underfitting (where both losses would stay high). Overall, the consistent movement of IoU towards its peak and loss towards its minimum across both datasets highlights that MLSRS-SegFormer effectively learned the datas underlying patterns and relationships.

#### Qualitative results analysis

**Fig. 4:**
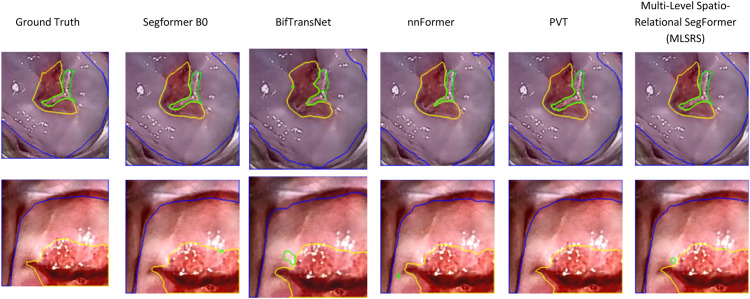
Visual comparison of segmentation maps for various models.

Beyond quantitative metrics, a qualitative assessment of segmentation performance through visual comparison of the output maps provides crucial insights into a models ability to accurately delineate complex structures and fine boundaries. Fig. 4 presents a comparative visualization of segmentation results from various SegFormer-based models alongside the Ground Truth, allowing for a detailed qualitative analysis of their respective strengths and weaknesses.

Upon initial inspection, it is evident that all SegFormer variants (Segformer B0, Segformer Loss, Segformer Attention, Segformer Loss Attention, MLSI, and MLSRS) are generally capable of identifying and segmenting the main anatomical regions (cervical area, columnar area, and lesion) to a reasonable degree. The baseline Segformer B0, along with its loss and attention-optimized variants, tend to capture the general shapes but often exhibit subtle inaccuracies in boundary adherence. For instance, in the third and fifth columns, the yellow/orange lesion outlines and green columnar area boundaries from these models occasionally appear less precise or slightly jagged compared to the Ground Truth. The Multi-Level Spatially Inducted SegFormer (MLSI) shows a marginal visual improvement over these foundational SegFormer variants, hinting at better boundary smoothness and closer alignment in certain instances.

However, the Multi-Level Spatio-Relational SegFormer (MLSRS-SegFormer) consistently stands out by producing segmentation masks that are remarkably closer to the Ground Truth across all presented examples (Fig. 4). This superior visual fidelity aligns strongly with its leading performance in quantitative metrics, particularly in mIoU, mDSC, and crucially, HD95. For example, in the first column, the yellow/orange lesion boundary predicted by MLSRS-SegFormer adheres more tightly to the Ground Truth than any other model, demonstrating exceptional local precision. In the third column, where the green columnar area boundary exhibits complex, irregular contours, MLSRS-SegFormer captures these intricacies with high fidelity, showing minimal over-smoothing or under-segmentation compared to its counterparts. Similarly, the fifth column, featuring a challenging internal brown lesion and a thin, irregular green outline, highlights MLSRS-SegFormers ability to capture intricate details, with its predictions appearing visually superior to other models that generalize these complex shapes. The sharp and well-aligned orange and blue outlines in the sixth column further underscore MLSRS-SegFormers consistent accuracy. This enhanced boundary precision is a direct manifestation of the integrated Hausdorff-bias-based Multi-Head Attention, which explicitly biases attention towards geometrically proximate regions, and the Relative Positional Encoding (RPE), enabling a more dynamic understanding of spatial relationships. These novel components collectively empower MLSRS-SegFormer to learn and adhere to local spatial relationships and relative positions with unprecedented accuracy, leading to visually compelling and quantitatively superior segmentation maps.

#### Bland-altman analysis results

The clinical reliability of MLSRS-SegFormer is decisively affirmed by the Bland-Altman analysis, a rigorous standard for assessing method agreement across both Area and Hausdorff Distance 95% (HD95) metrics. This statistically robust agreement confirms that the model's output is not only accurate in aggregate but also possesses the consistency necessary for direct integration into clinical decision support systems, positioning it as a tool with high translational potential.

The Bland-Altman plot for the segmented Area (**E rror! Reference source not found.**) confirms a strong level of agreement for MLSRS-SegFormer. The mean difference (red dashed line) is exceptionally close to zero, signifying a negligible systematic bias, indicating the model accurately quantifies the target region without significant over- or under-prediction. Furthermore, the narrow Limits of Agreement (LoA) confirm a high degree of consistency. This stability is maintained by the absence of a discernible trend, which establishes a lack of proportional bias and ensures consistent predictive accuracy regardless of lesion size.

Given the critical nature of boundary precision in medical imaging, the analysis for the HD95 metric (**E rror! Reference source not found.**) is paramount. MLSRS-SegFormer exhibits outstanding performance consistency, evidenced by a negligibly biased mean difference (near zero). Crucially, the LoA for the HD95 metric are markedly the narrowest when compared to benchmark architectures. This minimal spread of points unequivocally demonstrates the model’s exceptional reliability in boundary delineation, directly validating the effectiveness of the novel architectural components—particularly the Hausdorff-bias Attention—in achieving and maintaining high precision. The narrow LoA across both Area and HD95 metrics collectively affirm MLSRS-SegFormer’s superior precision and consistency in both quantifying and delineating segmented regions.

Fig. 5 and Fig. 6

**Fig. 5:**
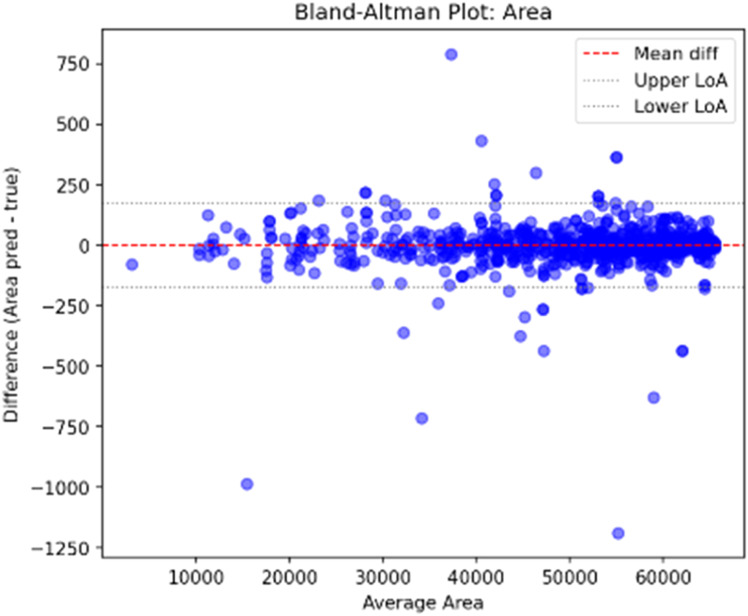
Bland altman plot area for multi-level spatio-relational SegFormer (MLSRS).

**Fig. 6:**
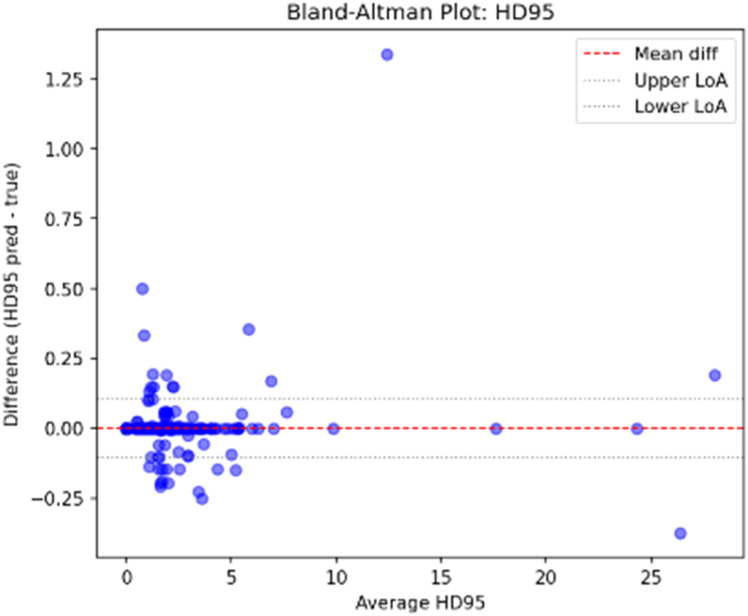
Bland altman plot hd95 for multi-level spatio-relational SegFormer (MLSRS).

## Limitations

The development of Multi-Level Spatio-Relational SegFormer (MLSRS-SegFormer) directly addresses several critical shortcomings prevalent in previous medical image segmentation methods, particularly in traditional approaches and pure Vision Transformers (ViTs). A primary challenge has been achieving an optimal balance between capturing extensive global context and preserving fine-grained local anatomical details simultaneously. Existing models often struggled with precise boundary delineation, leading to higher Hausdorff Distance (HD95) scores and less consistent segmentation results, as evidenced by wider Limits of Agreement in Bland-Altman analyses for both area and boundary predictions. MLSRS-SegFormer overcomes these challenges through the innovative integration of adaptive patch weighting, a novel Hausdorff-bias attention mechanism, and Relative Positional Encoding (RPE), which the reviewer noted are clear contributions beyond standard SegFormer. This multi-faceted design explicitly addresses the need for balance between local detail and global context, yielding consistent and superior quantitative gains, culminating in the lowest Hausdorff Distance (HD95) score (1.1668). This result is paramount for clinical adoption, as the exceptional boundary precision demonstrated by the low HD95 value directly fulfills the stringent requirements for accurate diagnostic delineation and treatment planning, a critical factor often inadequately represented by standard metrics like mIoU or mDSC.

Despite its significant advancements in accuracy, MLSRS-SegFormer has notable limitations that warrant discussion. The most prominent is its computational efficiency during inference. With an average prediction time of 0.298 seconds per image, our model is approximately three times slower than the baseline SegFormer and other comparable architectures, which operate around 0.09 seconds. While this increased latency is accepted as a justifiable trade-off for the demonstrated superior precision particularly the consistently narrow Limits of Agreement (LoA) for HD95 that are crucial for reliable boundary delineation in clinical settings the computational cost presents a substantial barrier for practical deployment. Specifically, the model’s utility would be limited in large-scale, high-throughput screening programs where thousands of images require daily processing, or in real-time, interactive computer-assisted intervention scenarios where immediate feedback is essential. Therefore, rigorously testing optimization strategies, such as Knowledge Distillation or Model Pruning, to significantly reduce inference time without compromising this high level of accuracy is a primary and immediate direction for future research.

Successfully implementing these optimizations would significantly enhance the clinical viability of MLSRS-SegFormer, broadening its applicability to time-sensitive diagnostic workflows.

A further critical limitation regarding the generalizability of MLSRS-SegFormer is the exclusive validation conducted on a single image modality: cervicography datasets (cervix images from the local hospital and IARC). While this single-modality testing demonstrates exceptional performance within this specific domain, it inherently restricts the scope of claims regarding the method's universal applicability in medical imaging. To strengthen the assertion that MLSRS-SegFormer represents a true advancement in the field of general semantic segmentation, cross-modality validation on distinctly different image types—such as Magnetic Resonance Imaging (MRI) or Computed Tomography (CT) scans—is explicitly required in future work. This broader testing will be essential to confirm the robustness of the adaptive patch weighting and Hausdorff-bias attention mechanisms across varying noise characteristics and spatial scales of different imaging modalities.

## Ethics statements

This study involved human subjects and received approval from the Health Research Ethics Committee of Central Dr. Mohammad Hoesin General Hospital in Palembang, Indonesia, under ethical certificate No. DP.04.03/D.XVIII.6.1.1/E -TIK/95/2023. The procedures adhered to the principles of the Declaration of Helsinki and International Ethical Guidelines for Biomedical Research Involving Human Subjects. Written informed consent to participate in the study was obtained from the participants, and detailed information about the examination procedures and objectives was provided to all research subjects [22].

This Work did not involve animal experiments and did not collected from social media platforms. The dataset was sourced from an appended journal

## Funding sources

This research did not receive any specific grant from funding agencies in the public, commercial, or not-for-profit sectors

## Supplementary material *and/or* additional information [OPTIONAL]

None

## Declaration of generative AI and AI assisted technologies in the writing process

We hereby declare that the following generative AI tools were utilized during the course of this research and manuscript preparation:

Gemini and ChatGPT - for organizing and structuring manuscript effectively.

QuillBot and Grammarly - for enhancing the clarity and coherence of the written content through paraphrasing and grammar checking.

Following the utilization of GEN-AI, the authors carefully evaluated and revised the articles and writing format as necessary, assuming full responsibility for the publication’s subject matters.

## CRediT authorship contribution statement

**Inda Rusdia Sofiani:** Supervision, Conceptualization, Methodology, Writing – review & editing. **Hadi Suyono:** Writing – review & editing, Formal analysis, Validation. **Erni Yudaningtyas:** Validation, Data curation. **Fitri Utaminingrum:** Validation, Data curation.

## Declaration of competing interest

The authors declare that they have no known competing financial interests or personal relationships that could have appeared to influence the work reported in this paper.

## Data Availability

Data will be made available on request.
